# Characterization of the Genome Feature and Toxic Capacity of a *Bacillus wiedmannii* Isolate From the Hydrothermal Field in Okinawa Trough

**DOI:** 10.3389/fcimb.2019.00370

**Published:** 2019-10-25

**Authors:** Yan Zhao, Chen Chen, Han-jie Gu, Jian Zhang, Li Sun

**Affiliations:** ^1^CAS Key Laboratory of Experimental Marine Biology, Center for Ocean Mega-Science, Institute of Oceanology, Chinese Academy of Sciences, Qingdao, China; ^2^Laboratory for Marine Biology and Biotechnology, Pilot National Laboratory for Marine Science and Technology (Qingdao), Qingdao, China; ^3^College of Earth and Planetary Sciences, University of Chinese Academy of Sciences, Beijing, China; ^4^Deep Sea Research Center, Institute of Oceanology, Chinese Academy of Sciences, Qingdao, China

**Keywords:** *Bacillus wiedmannii*, deep-sea hydrothermal vent, cytotoxicity, virulence, genome

## Abstract

The *Bacillus cereus* group is frequently isolated from soil, plants, food, and other environments. In this study, we report the first isolation and characterization of a *B. cereus* group member, *Bacillus wiedmannii* SR52, from the hydrothermal field in the Iheya Ridge of Okinawa Trough. SR52 was isolated from the gills of shrimp *Alvinocaris longirostris*, an invertebrate species found abundantly in the ecosystems of the hydrothermal vents, and is most closely related to *B. wiedmannii* FSL W8-0169. SR52 is aerobic, motile, and able to form endospores. SR52 can grow in NaCl concentrations up to 9%. SR52 has a circular chromosome of 5,448,361 bp and a plasmid of 137,592 bp, encoding 5,709 and 189 genes, respectively. The chromosome contains 297 putative virulence genes, including those encoding enterotoxins and hemolysins. Fourteen rRNA operons, 107 tRNAs, and 5 sRNAs are present in the chromosome, and 7 tRNAs are present in the plasmid. SR52 possesses 13 genomic islands (GIs), all on the chromosome. Comparing to FSL W8-0169, SR52 exhibits several streaking features in its genome, notably an exceedingly large number of non-coding RNAs and GIs. *In vivo* studies showed that following intramuscular injection into fish, SR52 was able to disseminate in tissues and cause mortality; when inoculated into mice, SR52 induced acute mortality and disseminated transiently in tissues. *In vitro* studies showed that SR52 possessed hemolytic activity, and the extracellular product of SR52 exhibited a strong cytotoxic effect. These results provided the first insight into the cytotoxicity and genomic feature of *B. wiedmannii* from the deep-sea hydrothermal environment.

## Introduction

The deep-sea environment is characterized by high pressure, low temperature (except in hydrothermal vents), and low concentrations of labile organic carbon, all of which modulate the structures of local microbial community (Nagata et al., 2010; Louvado et al., 2015). Deep-sea hydrothermal vents are localized areas of the seabed with high biological productivity contributed by the food webs of invertebrates and microbes (Van Dover, 2014). The population structure and phylogenetic diversity of microbes in deep-sea environments, particularly those associated with hydrothermal vents, have been investigated recently (Arakawa et al., 2006; Batzke et al., 2007; He et al., 2017; Meier et al., 2017; Zhang et al., 2017; Fortunato et al., 2018; Dick, 2019). Various bacteria, including *Proteobacteria, Actinobacteria*, and *Firmicutes*, as well as archaea and viruses have been detected in hydrothermal vent ecosystems (Moyer et al., 1995; Reysenbach et al., 2000; Ortmann and Suttle, 2005; Sylvan et al., 2012; Dick et al., 2013; He et al., 2017; Dick, 2019).

The *Bacillus cereus* group is a subdivision of the genus *Bacillus*. It comprises more than 20 closely related species including diverse Gram-positive heterotrophic aerobic and facultative anaerobic bacilli with the ability to form environmentally resistant and metabolically inert spores (Schmidt et al., 2011; Liu et al., 2015, 2017a). The *B. cereus* group is ubiquitously present in various environments including many types of soils, sediments, plants, water, and food (Stenfors Arnesen et al., 2008; Liu et al., 2017b). Recently, bacteria recovered from diverse marine environments were proposed to represent nine novel species of the *B. cereus* group based on polyphasic taxonomic analysis (Liu et al., 2017a). Several studies have indicated the presence of *Bacillus* species in the deep sea (Marteinsson et al., 1996; Liu et al., 2006; Kurata et al., 2015; Wen et al., 2015), but to our knowledge, no *B. cereus* group from deep sea hydrothermal vents has been reported.

Members of the *B. cereus* group are known to produce numerous enzymes and metabolites and serve as probiotics for animal and plant growth; however, most studies on this group have been focused on the aspects of epidemiology and pathogenesis (Rasko et al., 2005; Chang et al., 2007; Kevany et al., 2009; Gisbert et al., 2013; Liu et al., 2015). *Bacillus anthracis* is most famous for its potential to cause the severe disease anthrax, genetically determined by its plasmids pXO1 and pXO2 (Dixon et al., 1999). Similarly, *Bacillus thuringiensis* produces insecticidal parasporal protein crystals (Cry) and/or cytolytic (Cyt) proteins that are mostly encoded on plasmids (Palma et al., 2014). *Bacillus cytotoxicus* was recently described as a thermotolerant member of the *B. cereus* group characterized by the production of cytotoxin K-1 (CytK-1) (Guinebretiere et al., 2013). *Bacillus cereus*, which produces a heat stable peptide toxin named cereulide and three enterotoxins, i.e., non-hemolytic enterotoxin (Nhe), hemolysin BL(Hbl), and cytotoxin K (CytK), is a common cause of foodborne infectious disease and food poisoning characterized by symptoms of diarrhea or vomiting (Stenfors Arnesen et al., 2008). In addition, serious opportunistic non-gastrointestinal diseases have been attributed to *B. cereus*, such as septicemia, endophthalmitis, pneumonia, endocarditis, meningitis, and encephalitis (Bottone, 2010). Four other members of the *B. cereus* group, i.e., *Bacillus mycoides, Bacillus pseudomycoides, Bacillus wiedmannii*, and *Bacillus weihenstephanensis*, may also cause food poisoning (Nakamura, 1998; Hendriksen et al., 2006; Mei et al., 2014; Miller et al., 2016).

In this study, we reported for the first time the identification of a *B. weidmannii* strain, SR52, associated with animal inhabitants of the hydrothermal vent in Okinawa Trough. We analyzed the biological, genomic, and potential infective features of SR52. Our results provide the first insight into the genetic property and toxicity of deep-sea *B. cereus*.

## Materials and Methods

### Ethics Statement

Live animal studies were approved by the Ethics Committee of Institute of Oceanology, Chinese Academy of Sciences. All of the methods were carried out in accordance with the relevant guidelines.

### Isolation of SR52

SR52 was isolated from the shrimp *Alvinocaris longirostris* collected at the Iheya Ridge hydrothermal vent field of Okinawa Trough (Sun et al., 2016). The shrimp were collected with a carousel sample collector carried on a remotely operated vehicle (ROV) (Specialist Machine Developments Limited, Northumberland, UK) and thoroughly washed with sterile seawater. For bacterial isolation, the gills were immediately removed from the shrimp and homogenized in PBS. The homogenate was plated on marine agar 2216E medium (Sun et al., 2015), and the plates were incubated at 28°C for 2–3 days under aerobic conditions. The colonies on the plates were screened according to their shape, size, margin, color, and opacity (Valiente Moro et al., 2013). Colonies of each type were selected and purified by re-culturing. The purified isolates were resuspended in marine 2216E medium containing 15% (v/v) glycerol and stored at −80°C. The species identities of the isolated bacteria were determined based on 16S rRNA gene sequence as reported previously (Sun et al., 2015). A total of 30 isolates were obtained, including three isolates belonging to the genus *Bacillus* and one isolate belonging to *B. cereus* group, which was named SR52.

### Morphological Features and Growth Characteristics of Strain SR52

Strain SR52 was cultured at 28°C overnight in 2216E medium and resuspended in PBS (Hyclone, Logan, UT, USA); the morphology of the bacterial cells was observed using a transmission electron microscope (HT7700, Hitachi, Tokyo, Japan). Sporulation analysis was carried out by growing SR52 at 28°C for 2–3 days in 2216E medium containing 5 mg/L MnSO_4_. The spores were stained with carbol fuchsin using a Spore Stain Kit (Solarbio, Beijing, China) according to manufacturer's instruction, followed by observation with a microscope (Ti-S/L100, Nikon, Tokyo, Japan). The growth temperature was determined by culturing SR52 at 5, 10, 15, 20, 25, 30, 35, 40, and 45°C in marine 2216E medium with shaking for up to 3 days. The sodium tolerance was determined by culturing SR52 in 2216E medium containing different concentrations of NaCl (0–12%, w/v, with increments of 1.0%). The pH range of growth was examined by culturing SR52 in 2216E medium with the appropriate biological buffers (pH 4–10, with increments of 0.5 pH units) at 28°C. The motility of SR52 was assayed as reported previously (Gu et al., 2019).

### Animal and Cell Culture

Clinically healthy turbot (*Scophthalmus maximus*, 13.2 ± 2.4 g) were purchased from a local fish farm and maintained at 20°C in aerated seawater. Fish were acclimatized in the laboratory for 2 weeks before experimental manipulation. Before the experiment, the fish were verified to be pathogen free as described previously (Zhang et al., 2012). BALB/c mice (female, 8–10 weeks, and 14 ± 2 g) were purchased from Qingdao Daren Fortune Animal Technology Co., Ltd (Qingdao, China). For tissue collection, fish were euthanized with tricaine methanesulfonate (Sigma, St. Louis, MO, USA) as described previously (Wang et al., 2009). The mice were anesthetized with ketamine (80 mg/kg) (Ketavet, Pfizer, Berlin, Germany) (Dietert et al., 2017). RAW264.7, a murine monocyte-macrophage cell line, was purchased from American Tissue Culture Collection (ATCC, Manassas, VA, USA). The cells were cultured in Dulbecco's minimal Eagle's medium (DMEM) (Gibco, Carlsbad, CA, USA) containing 10% fetal bovine serum (FBS) (Gibco, Carlsbad, CA, USA) at 37°C with 5% CO_2_. Turbot peripheral blood leukocytes (PBL) were prepared and cultured based on the method reported previously (Li and Sun, 2018).

### Phylogenetic Analysis

For phylogenetic analysis, 23 16S rRNA genes and 10 partial *rpoB* genes (632 bp, from 2,455 to 3,086) (Huck et al., 2007) from SR52 and other *Bacillus* sp. strains were obtained (Table S1). Phylogenetic trees were constructed via the neighbor-joining (NJ) method (Saitou and Nei, 1987) and the maximum likelihood method (Felsenstein, 1981) for 16S rRNA gene and partial *rpoB* gene, respectively, with 1,000 bootstrap replicates using MEGA 5.0. The average nucleotide identity (ANI) was calculated using the EzBiocloud web service(https://www.ezbiocloud.net/tools/ani).

### DNA–DNA Hybridization (DDH)

DDH was carried out between strain SR52 and other representative strains of *B. cereus* group species and analyzed using genome-to-genome distance calculator (GGDC2.1) (https://ggdc.dsmz.de/ggdc.php; Meier-Kolthoff et al., 2013). All of the predicted pairwise DDH values were obtained under the recommended Formula 2 (Auch et al., 2010; Meier-Kolthoff et al., 2013). The genome sequences of the *B. cereus* group strains used in DDH were downloaded from NCBI (NCBI accession numbers are shown in Table S1).

### Genome Sequencing and Analysis

Total genomic DNA of strain SR52 was extracted using E.Z.N.A.^®^ Bacterial DNA Kit (Omega Bio-Tek, Doraville, USA) according to the manufacturer's instructions. The total DNA obtained was subjected to quality control by agarose gel electrophoresis and quantified by Qubit. The SR52 genome was sequenced by Single Molecule, Real-Time (SMRT) technology. Genome sequencing was conducted by Novogene Bioinformatics Technology Co., Ltd. (Beijing, China) using the third-generation PacBio RSII platform (Pacific Biosciences, Menlo Park, USA). After sequencing, the low-quality reads were filtered out and assembled to generate a single contig without gaps using SMRT portal assembly software (Berlin et al., 2015). The genome sequence data of SR52 have been deposited in GenBank under the accession numbers of CP032365 (chromosome) and CP032366 (plasmid). Transfer RNA (tRNA) genes were predicted with tRNAscan-SE (Lowe and Eddy, 1997), ribosome RNA (rRNA) genes were predicted with rRNAmmer (Lagesen et al., 2007), and sRNAs were predicted using BLAST against thr Rfam (Gardner et al., 2009) database. Repetitive sequences were predicted using RepeatMasker (Saha et al., 2008). Tandem repeats were analyzed using TandemRepeatFinder (Benson, 1999). Genomic islands were predicted with IslandPath-DIOMB (Hsiao et al., 2003). Gene prediction was performed on SR52 genome using GeneMarkS software (http://topaz.gatech.edu/GeneMark) with an integrated model combining the GeneMarkS generated (native) and Heuristic model parameters (Besemer et al., 2001). A whole genome Blast (Altschul et al., 1990) search (*E*-value <1 × e^−5^, minimal alignment length percentage larger than 40%) was performed against six databases, i.e., Kyoto Encyclopedia of Genes and Genomes (KEGG), Clusters of Orthologous Groups (COG), Non-Redundant Protein Database databases (NR), Swiss-Prot, Gene Ontology (GO), and TrEMBL. Pathogenicity analysis was conducted via a whole genome Blast search of Virulence Factors of Pathogenic Bacteria database (VFDB) (Chen et al., 2012). Genome overview was performed by Circos to show annotation information (Krzywinski et al., 2009). The analysis of specific genes and orthologous genes were conducted using the CD-HIT software (Version 4.6.1) with a threshold of 50% pairwise identity and 0.7 length difference cutoff in amino acid.

### *In vivo* Infection

The median lethal dose (LD50) was determined as reported previously (Gu et al., 2019). For *in vivo* infection, SR52 was cultured in 2216E medium to an OD_600_ of 0.8, and the cells were washed and resuspended in PBS to 1 × 10^7^ CFU/ml. Turbot were challenged with an intramuscular (i.m.) injection of 100 μl SR52 suspension or an equal volume of PBS (control). Bacterial dissemination in tissues was determined as reported previously (Li et al., 2017). Briefly, at 12, 24, and 48 h post inoculation (hpi), liver, kidney, and spleen were aseptically removed from the fish (5 fish/time point) and homogenized in PBS. The homogenates were serially diluted and plated in triplicate on 2216E agar plates. The plates were incubated at 28°C for 24 h, and the colonies appearing on the plates were counted. The genetic nature of the colonies was verified by PCR. *In vivo* infection of mice was performed by inoculation via intraperitoneal (i.p.) injection of SR52 at the dose of 6 × 10^6^ CFU/animal.

### Cytotoxicity and Hemolytic Activity

The strain SR52 was cultured as above, and cell-free culture supernatant was obtained by centrifugation (10,000 g, 10 min) and filtration through 0.22-μm-pore-size Millipore filters (Millex^®^-GP, Cork, Ireland). For cytotoxicity analysis, PBL were resuspended in L-15 medium containing 10% fetal bovine serum (FBS) (Cibco, Carlsbad, CA, USA) to 5 × 10^6^ cells/ml. PBL was added to 35 mm confocal dishes (1 ml/dish) and treated with either SR52 supernatant (10%, v/v) or 2216E medium (10%, v/v) for 30 min at 22°C. Scanning electron microscopy (SEM) was performed as reported previously (Chi and Sun, 2016). For fluorescent microscopy, Sytox Green was added to the cells, followed by incubation for 5 min, and the cells were then observed with a confocal microscope (LSM 710, Zeiss, Jena, Germany).

The hemolytic activity assay was performed by culturing by culturing SR52 and *B. subtilis* subsp. subtilis 168 (CGMCC accession No. 1.1390) in marine 2216E medium to an OD_600_ of 0.8. The cells were then used for the preparation of cell-free culture supernatant as described above and for the preparation of bacterial suspension as follows: the cells were centrifuged, washed, and resuspended in PBS as above to 2 × 10^8^ CFU/ml. The hemolytic assay was performed by adding 50 μl of SR52, strain 168, SR52 supernatant, strain 168 supernatant, or PBS into Oxford cups placed on a 4% rabbit blood agar plate (Hope Bio, Qingdao, China). The plate was incubated at 28°C overnight and observed for hemolytic halos.

Intracellular infection assay was performed as reported previously (Sui et al., 2017).

## Results

### Morphology and Growth of SR52

**Figure 1:**
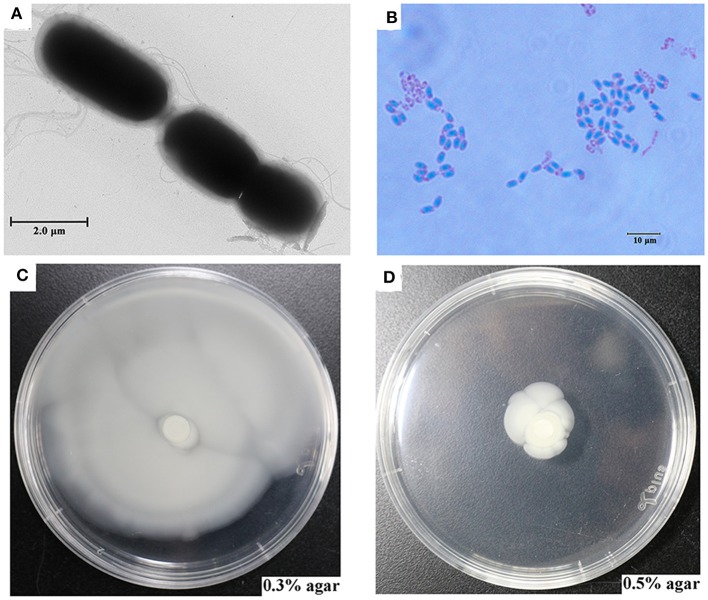
Morphology and motility of SR52. **(A)** SR52 observed with a transmission electron microscope. **(B)** Micrograph of carbol fuchsin-stained spores of SR52. **(C,D)** SR52 suspension was spotted onto a marine 2216E plate containing 0.3 or 0.5% (w/v) agar, and bacterial growth was observed after 24 h incubation.

Strain SR52 was isolated from the gills of the shrimp *A. longirostris* collected from the Iheya Ridge hydrothermal field of Okinawa Trough. Transmission electron microscopy (TEM) examination showed that SR52 was rod-shaped and motile with peritrichous flagella (Figure 1A). SR52 produced endospores, which were oval and located centrally in the cell (Figure 1B). SR52 grew at 5–40°C and pH 5–9.5 in the presence of up to 9.0% NaCl. The optimum NaCl concentration of SR52 was 1%, which is similar to that reported for other marine bacteria (Liu et al., 2017a). SR52 showed robust and moderate growth in 0.3 and 0.5% agar, respectively (Figures 1C,D).

### Phylogenetic Classification of SR52

**Figure 2:**
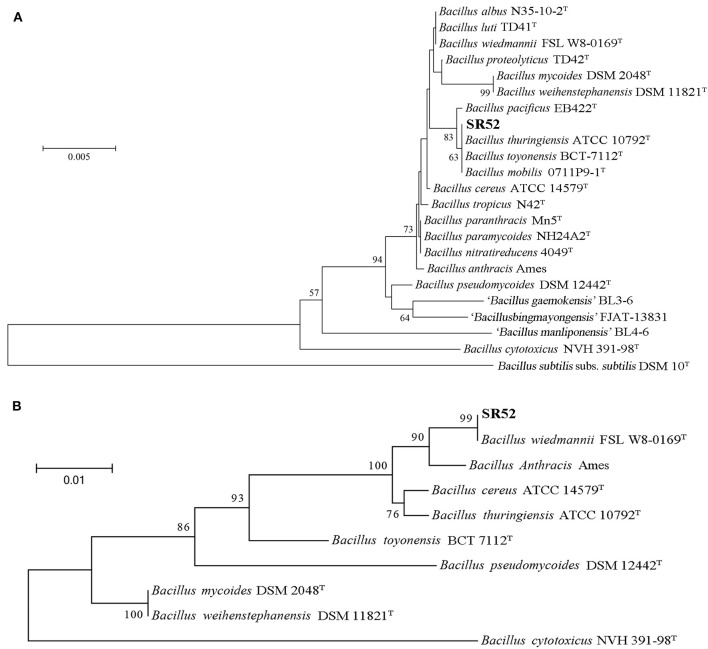
Phylogenetic analysis of SR52. **(A)** The neighbor-joining (NJ) tree was based on the 16S rRNA gene sequences of SR52 and the type strains of *Bacillus cereus* group. The type strain of the type species, DSM 10, was used as an outgroup. Bootstrap values >50% are shown at branch points. The bar represents 0.005 nucleotide substitution rate (K_nuc_). **(B)** The maximum likelihood tree was constructed based on *rpoB* sequences using MEGA version 5.0, and 1,000 replications of Bootstrap analysis were used to estimate the confidence level of the tree topologies. Bootstrap values >70 are displayed on branches. The bar represents 0.01 substitutions per site.

Analysis of 16S rRNA sequence indicated that SR52 was closely related to the *Bacillus* species in the *B. cereus* group (Figure 2A). A more discriminatory analysis based on the *rpoB* gene showed that SR52 formed a coherent cluster with *B. wiedmannii* FSL W8-0169 (Figure 2B), which was proposed as a novel species belonging to the *B. cereus* group recently (Miller et al., 2016). The *rpoB* gene phylogeny was confirmed via Genome-to-Genome Distance Calculator (GGDC) analysis. Predicted pairwise DDH values of SR52 and representative strains of *B. cereus* group species, except for *B. wiedmannii* FSL W8-0169, are substantially below the species cut-off 70% (Richter and Rosselló-Móra, 2009). The DDH value between SR52 and *B. wiedmannii* FSL W8-0169 was 88.4% (Table S2). The average nucleotide identity (ANI) between SR52 and FSL W8-0169 was 96.2%, which is higher than the species demarcation threshold 95% (Richter and Rosselló-Móra, 2009). These results indicate that SR52 is a member of the *B. wiedmannii* species.

### Genome Features of SR52

**Figure 3:**
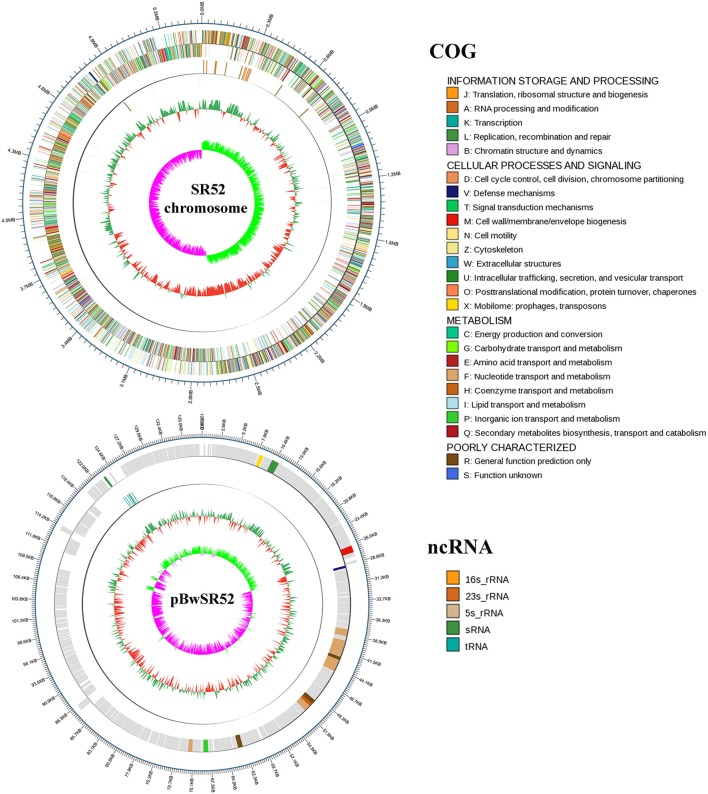
Circular maps of SR52 chromosome and plasmid pBwSR52. The base pairs are indicated outside the outer circle (Circle 1). Circle 1 represents the coding genes colored according to their functional annotations based on the COG database, with the forward strand on the outside and the reverse strand on the inside. Circle 2, noncoding RNAs (ncRNA); Circle 3, GC content; Circle 4, GC skew+ (green) and GC skew– (purple).

The complete genome sequence of SR52 was obtained. SR52 possesses a circular chromosome consisting of 5,448,361 bp with an average G+C content of 35.42% and a circular plasmid (named pBwSR52) consisting of 137,592 bp with an average G+C content of 36.81% (Figure 3; Table 1). The coding region accounts for 84.63% of the chromosome and includes 5,709 genes with an average length of 808 bp, while the plasmid pBwSR52 has a coding percentage of 87.74% and comprises 189 genes with an average length of 639 bp. The majority of genes are encoded in the leading strand, with a strong bias in gene orientation (Figure 3). A total of 42 rRNAs (grouped into 14 operons), 107 tRNAs, and 5 sRNAs were found in the chromosome, while 7 tRNAs were found in the plasmid. SR52 possesses 13 genomic islands (GIs), all located on the chromosome, with an average size of 11 kb. The GC content of the GIs ranges from 31.1 to 41.8%; however, most of the GC content is lower than that of the chromosome (35.42%; Table S3). Some of the GIs encode proteins related to spores, flagellae, cell membranes/walls, DNA methylation, and antibody-resistance. The SR52 genome contains 222 and 12 interspersed repeats in the chromosome and plasmid, respectively, with an average length of 86 and 83 bp, respectively (Table S4). These repeats fall into five types, i.e., LTR, DNA, LINE, SINE, and RC (Jurka et al., 2007; Saha et al., 2008). In addition, 317 and 7 tandem repeats were identified in the chromosome and plasmid, respectively, with repeat length of 3–837 and 12–39 bases, respectively.

**Table 1:** Feature of SR52 and W8-0169.

**Feature**	**SR52**			**W8-0169**
	**Chromosome**	**Plasmid**	**Total**	**Genome**
Size (bp)	5,448,361	137,592	5,585,953	5,337,981
Number of genes	5,709	189	5,898	5,428
Coding region (%)	84.63	87.74	84.7	84.1
Average gene length (bp)	808	639	802	827
G+C content (%)	35.42	36.81	35.46	35.19
rRNAs	14 × (16S-23S-5S)	0	14 × (16S-23S-5S)	1 × (16S-23S-5S)
tRNAs	107	7	114	63
sRNAs	5	0	5	9
Genomic islands	13	0	13	0
Tandem repeats	317	7	324	309
Interspersed repeats	222	12	234	204
Virulence genes	297	0	297	284

### Comparative Genome Analysis of SR52 and FSL W8-0169

The genomes of SR52 and *B. wiedmannii* FSL W8-0169, which is most closely related to SR52, were compared (Table 1). The chromosome of SR52 was comparable in size and GC content to that of FSL W8-0169; however, SR52 carries a plasmid (pBwSR52) which is absent in FSL W8-0169. The number of predicted genes in SR52 (5,898) is 8.65% more than that in FSL W8-0169 (5,428), but the average gene length is slightly shorter in SR52 (802 bp) than in FSL W8-0169 (827 bp). SR52 and FSL W8-0169 share 4,609 orthologous genes, and contain 897 and 381 specific genes, respectively. Strikingly, of the 189 genes in the plasmid of SR52, 187 genes have no orthologs in FSL W8-0169. Most of the specific genes of SR52 were annotated as hypothetical proteins with unknown functions. In addition to the specific protein-coding genes, a significant difference in ncRNA (rRNA, sRNA, and tRNA) was observed between SR52 and FSL W8-0169. Compared with FSL W8-0169, which has a single rRNA operon, 63 tRNAs, and 9 sRNAs, SR52 contains 14 rRNA operons, 114 tRNAs, and 5 sRNAs. A marked difference was also observed in GIs between SR52 and FSL W8-0169. In sharp contrast to FSL W8-0169, which has no GI, SR52 contains 13 GIs. The numbers of repeat sequences, including tandem repeats and interspersed repeats, are similar in these two strains. There are 297 predicted virulence genes in SR52 (Table S5), which are similar in number to the predicted virulence genes in FSL W8-0169 (284). Most of the virulence genes are shared by the two strains. Thirty of the 297 predicted virulence genes of SR52 are absent in FSL W8-0169, and most of these SR52-specific virulence genes are related to capsule and flagella (Table S5).

### Putative Virulence-Associated Genes in SR52 Genome

It is known that *Bacillus* species have a high capacity to secrete proteins, including virulent toxins, into the surrounding environment (Stenfors Arnesen et al., 2008). Several genes encoding Sec-dependent protein export pathway components were identified in the SR52 genome: *secA* (SR52-GM005594), *secD/F* (SR52-GM004836), *secE* (SR52-GM000296), *secG* (SR52-GM005537), *yajC* (SR52-GM004842), *yidC* (SR52-GM005462 and SR52-GM005896), *ffh* (SR52-GM004197), and *ftsY* (SR52-GM004199). Two genes of the twin-arginine translocation (Tat) system, *tatA* (SR52-GM002370) and *tatC* (SR52-GM002371), and five type I signal peptidase (SPase) genes (SR52-GM003267, SR52-GM003281, SR52-GM004191, SR52-GM000628, and SR52-GM001311) and one type II SPase (SR52-GM004248) occur in SR52. Many genes associated with secretion and virulence in *B. cereus* are known to be regulated by the transcriptional activator PlcR (Slamti and Lereclus, 2002). Analysis of the *plcR* (SR52-GM005764) gene sequence of SR52 indicated the absence of the nonsense mutations in the *plcR* of *B. anthracis*. A *papR* gene (SR52-GM005763) located 86 bp downstream of *plcR* was found, and, based on the feature of the last five C-terminal residues of the encoded PapR (Slamti and Lereclus, 2005), PapR should be able to activate the *plcR* regulon in SR52. There are two putative gene clusters (SR52-GM003972 to SR52-GM003986 and SR52-GM005677 to SR52-GM005688) encoding capsule and one putative gene cluster (SR52-GM001787 to SR52-GM001833) encoding flagella in the genome of SR52.

BLAST analysis against the Virulence Factor Database (VFDB) revealed that SR52 possesses two hemolysin BL gene clusters, i.e., *hblCDA* (SR52-GM002662 to SR52-GM002664) and *hblCDAB* (SR52-GM002515 to SR52-GM002518) clusters, two hemolysin III (*hlyIII*) coding units (SR52-GM002369 and SR52-GM005862), and one non-hemolytic enterotoxin gene cluster (*nheABC*) (SR52-GM002001 to SR52-GM002003), the latter encoding the enterotoxin Nhe. SR52 contains three internalin genes (*inlA*) (SR52-GM000708, SR52-GM001495, and SR52-GM001496), which may facilitate the initial host cell invasion (Dramsi et al., 1997; Fedhila et al., 2006), and a sphingomyelinase gene (*sph*) (SR52-GM000833), which is involved in cytotoxicity and pathogenicity (Doll et al., 2013). In addition, three genes annotated as putative immune inhibitor A (*inhA*) (SR52-GM000827, SR52-GM001449, and SR52-GM003173), which are associated with bacterial virulence (Fedhila et al., 2002), were also detected in SR52.

### Toxicity of SR52 to Teleost Fish and Mice

**Figure 4:**
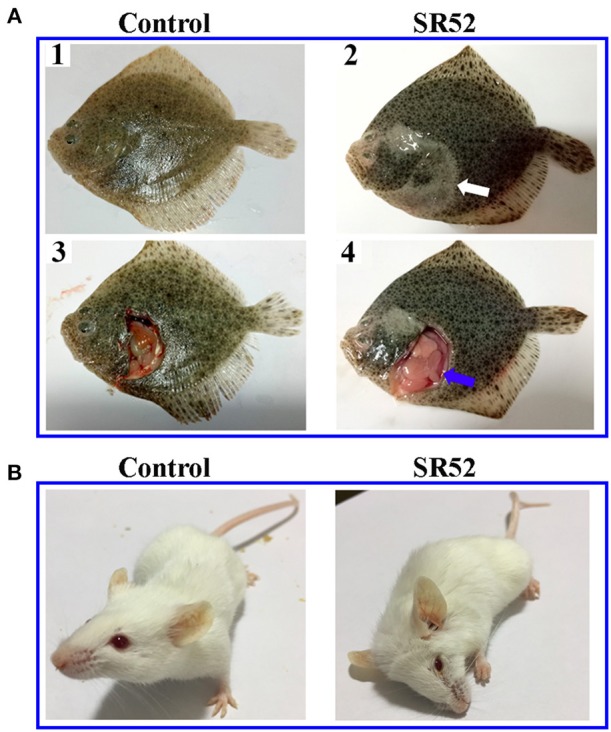
Clinical effects of SR52 on turbot and mice. **(A)** Turbot were inoculated with (right) or without (left; control) SR52 for 24 h and then observed for clinical signs. The upper panel (A1 and A2) shows the intact fish, with the white arrow indicating ulcer-like skin lesions; the lower panel (A3 and A4) shows the fish with some interior organs exposed, and the blue arrow indicates congestion of visceral organs. **(B)** Mice were inoculated with (right) or without (left; control) SR52 for 12 h and then observed for clinical signs. The SR52-injected mouse was in a moribund state.

**Figure 5:**
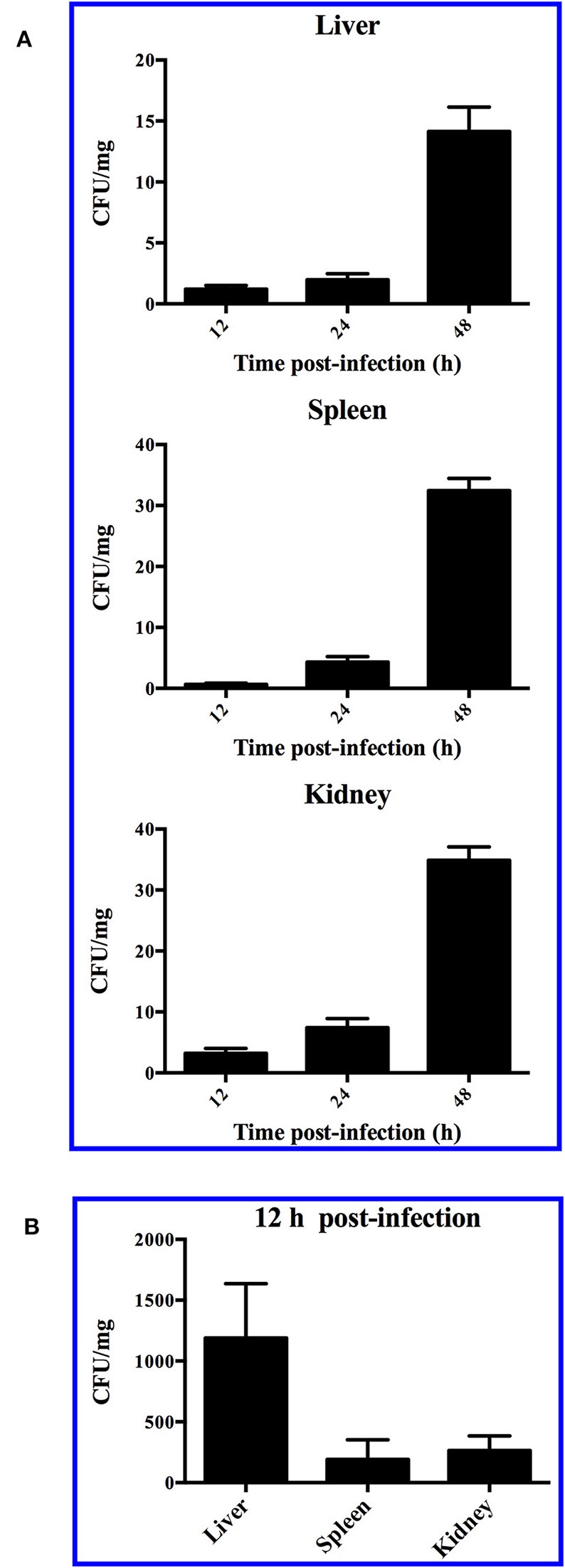
Dissemination of SR52 in fish and mouse tissues. Turbot **(A)** and mice **(B)** were inoculated with SR52, and bacterial recovery from the tissues was determined at different time points. The results are the means of triplicate experiments and shown as means ± SEM.

When turbot were immersed in sea water containing 1 × 10^8^ CFU/ml SR52, no mortality or clinical symptom was observed. When turbot were inoculated with SR52 via intramuscular (i.m.) injection, an LD_50_ value of 1.3 × 10^5^ CFU was obtained. Fish inoculated with SR52 exhibited ulcer-like skin lesions, ascites, and congestion of visceral organs (Figure 4). At 12, 24, and 48 h post-inoculation (hpi), SR52 dissemination into and colonization of the liver, spleen, and kidney of turbot were observed, with bacterial numbers increasing with time (Figure 5A). When mice were inoculated intraperitoneally (i.p.) with SR52, an LD_50_ value of 1.4 × 10^7^ CFU was obtained. At the dose of 1.2 × 10^6^ CFU, no mortality was observed. At the dose of 6 × 10^6^ CFU, 30% mortality was observed at 12 hpi, and no further mortality occurred thereafter. SR52-inoculated mice exhibited no apparent tissue lesions but showed reduced movement and weakness. At 12 hpi, SR52 was recovered from the liver, spleen, and kidney of moribund mice (Figure 5B), but no bacteria were recovered from the surviving mice at 24 and 48 hpi.

### Intracellular Infection Potential of SR52

To examine whether SR52 was able to invade into and survive in host cells, RAW264.7 cells, a mouse cell line, were incubated with SR52 at different multiplicities of infection (MOI) (1 and 10), and subsequent plate count revealed no intracellular SR52. A similar negative observation was made when the assay was performed with fish (turbot) PBL.

### Cytotoxic Effect of the Extracellular Product of SR52

**Figure 6:**
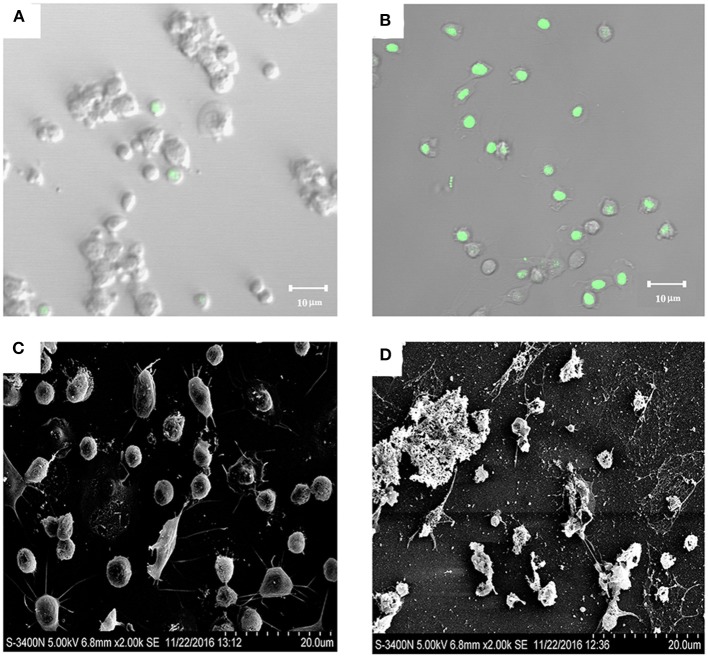
Cytotoxic effect of SR52 culture supernatant. Turbot peripheral blood leukocytes (PBL) were treated with **(B,D)** or without **(A,C)** the culture supernatant of SR52. The cells were then stained with Sytox Green and observed with a confocal microscope **(A,B)** or observed directly with a scanning electron microscope **(C,D)**.

Because SR52 caused no apparent intracellular infection, we further investigated whether the extracellular product of SR52 had any toxic effect on host cells. After incubation of turbot PBL with the culture supernatant of SR52, most cells stained positive with Sytox Green (Figures 6A,B), a fluorescent dye that is excluded from healthy cells with intact plasma membranes but enters cells if pores of sufficient size occur in the plasma membrane. Scanning electron microscopy revealed that treatment of turbot PBL with SR52 culture supernatant induced severe damage to cellular structure (Figures 6C,D). A similar cellular damaging effect of SR52 supernatant on RAW264.7 cells was also observed (data not shown).

### Hemolytic Effect of SR52

**Figure 7:**
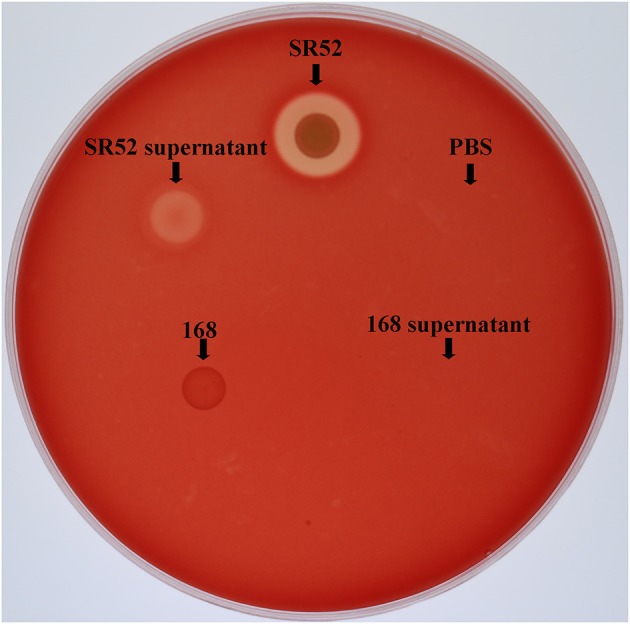
Hemolytic activity of SR52. SR52, the supernatant of SR52 culture, *Bacillus subtilis* subsp. *subtilis* 168, the supernatant of 168 culture, and PBS were transferred into Oxford cups placed on a rabbit blood agar plate, and the plate was monitored for hemolytic halo after overnight incubation.

Since SR52 carries hemolysin genes in the genome, we examined the hemolytic potential of SR52. The results showed that SR52 induced apparent lysis of rabbit red blood cells (Figure 7). A weaker but distinct lysis was also observed with SR52 culture supernatant. In contrast, *Bacillus subtilis* subsp. *subtilis* 168, a non-hemolytic bacterium, displayed no hemolytic activity.

## Discussion

Organisms of the *B. cereus* group are the subject of growing interest due to their potential economic, medicinal, and biodefense applications (Rasko et al., 2005; Stenfors Arnesen et al., 2008). In this study, we reported the first characterization of a *B. cereus* group member, SR52, from the hydrothermal field in Okinawa Trough. Combined analyses of 16S rRNA sequence and *rpoB* gene, as well as using GGDC indicated that SR52 is closely related to the *B. cereus* group and belongs to *B. wiedmannii*, members of which were isolated from dairy food and dairy environment and proposed recently as a novel species distinct from other species in the *B. cereus* group (Miller et al., 2016). A previous study showed that seven species in the *B. cereus* group collected from diverse marine environments tolerated salt concentration as high as 5–9%, whereas the *B. cereus* group members of terrestrial sources generally fail to grow when the salt concentration becomes higher than 5% (Liu et al., 2017a). Similarly, we found that consistent with its deep-sea source of isolation, SR52 was able to grow in 9.0% NaCl. Comparative genomic analysis of SR52 and the representative strain of *B. wiedmannii*, i.e., FSL W8-0169, revealed that SR52 harbors a markedly increased number of ncRNAs. Non-coding RNAs, mainly referring to rRNA, tRNA, and sRNA in bacterium, are essential for many cellular processes such as regulation of gene expression, RNA processing, and protein synthesis and secretion (Storz, 2002). Compared with FSL W8-0169, SR52 has 14 times more rRNA operons and almost twice the number of tRNAs. The number of tRNAs in SR52 (114) is higher than that (54–107) in 30 *B. wiedmannii* strains randomly selected from the NCBI data. These results suggested a more robust and/or sophisticated protein expression and synthesis system in SR52, which may facilitate its survival under the changing environmental conditions of the hydrothermal filed. Notably, while FSL W8-0169 has no GI, SR52 possesses 13 GIs encoding proteins related to spore, flagellar, cell membrane/wall, DNA methylation, and antibody resistance. Previous studies showed that in virulent bacteria, pathogenicity islands, which are a subclass of GI, contribute to rapid changes in virulence potential. In non-pathogenic bacteria, GIs are important for bacterial evolution by influencing traits, such as antibiotic resistance, symbiosis and fitness, and adaptation (Hacker and Carniel, 2001; Dobrindt et al., 2004; Gal-Mor and Finlay, 2006). The large amount of GIs in SR52 may be acquired by SR52 through horizontal gene transfer in the deep-sea environment, which is in line with the presence of a plasmid in SR52. These results suggest SR52 possessing the capacity to gain genetic elements from the environment. Given their coding information, the GIs of SR52 likely contribute to bacterial evolution and adaption to the hydrothermal environment by affecting survival under the specific condition.

The *in vivo* study in a turbot model showed that SR52 failed to invade fish via natural route (immersion) of infection, but induced mortality following i.m. injection, and that once being injected into fish, SR52 was able to disseminate in multiple tissues. These results indicate an ability of SR52 to survive *in vivo*. Previous studies involving mouse models showed that *B. cereus* infection led to overt inflammation that may drive immunopathology and septic shock in mice, resulting in rapid lethality, similar to LPS-induced endotoxaemia (Man et al., 2017; Mathur et al., 2019). In our study, when SR52 was inoculated into mice at the dose of 6 × 10^6^ CFU, acute mortality occurred within 12 h, and the mice exhibited clinical signs (quietness and motionlessness) akin to that reported by Burdon et al. (1967) in *B. cereus*-infected mice. The acute mortality-inducing capacity observed with SR52 is similar to that observed with another deep-sea hydrothermal vent isolate, *B. subtilis* G7, which caused rapid mortality in mice at the dose of 2.5 × 10^6^ CFU/g (corresponding to ~3.5 × 10^7^ CFU/mouse), whereas *B. subtilis* subsp. *subtilis* 168, a non-pathogenic strain, induced no mortality in mice at the same dose (Gu et al., 2019). Tissue dissemination of SR2 in mice was observed only during the early hours (12 h) of inoculation and was undetectable at 24 h in survived mice, suggesting a rapid clearance of SR2 by the host.

It is well-known that enterotoxins play a vital role in the virulence of pathogenic *B. cereus* species, and three cytotoxins, i.e., CytK, Hbl, and Nhe, are currently linked to *B. cereus*-induced diarrhea and cytotoxicity (Stenfors Arnesen et al., 2008; Bottone, 2010). However, several studies showed that only the Nhe-coding gene was found in all known strains of the *B. cereus* group, whereas the *hbl* and *cytK* genes occurred in <50% of strains tested randomly and often absent even from *B. cereus* strains isolated from disease outbreaks (Ehling-Schulz et al., 2005; Moravek et al., 2006; Bohm et al., 2015). Although Nhe was considered a dominant virulent factor in *B. cereus* (Moravek et al., 2006), multiple toxins appeared to act synergistically to cause cytotoxicity and disease (Stenfors Arnesen et al., 2008). In our study, both Hbl- and Nhe-encoding gene clusters were found in SR52 genome, indicating a potential for cytotoxicity and virulence. Furthermore, the two homologous *hbl* and *hbl*_*a*_ operons, the latter rarely occurring in *B. cereus* group strains (Bohm et al., 2015), are present in SR52. However, a previous report showed no significant difference between HBL and HBL_a_ in hemolytic and vascular permeability activity (Beecher and Wong, 2000). The genes encoding the β-barrel pore-forming enterotoxins CytK and hemolysin II are absent in SR52, whereas SR52 possesses another hemolytic toxin, hemolysin III. In addition to *hbl* and *nhe*, other toxin-associated genes were also detected in SR52, including *inhA, sph*, and *inlA*. In line with the presence of cytotoxin and hemolysin genes in its genome, SR52 showed apparent hemolytic effect, and the extracellular product of SR52 induced rapid destruction of fish and mouse cells, suggesting actual release of extracellular toxins and hemolysins by SR52. Similar observations have been made with strain FSL W8-0169 (Miller et al., 2016). To date, only one study concerning the virulence potential of FSL W8-0169 has been documented, in which it was shown that the culture supernatant of FSL W8-0169 was cytotoxic to HeLa cells (Miller et al., 2016). The similar cytotoxic properties of SR52 and FSL W8-0169 are in line with the fact that the predicted virulence genes of these two strains are highly similar. It is likely that the various cytotoxins and hemolysins may synergistically contribute to the tissue damage and lethal effects of SR52. Interestingly, in SR52, no toxin-encoding gene is located in the plasmid, which is in contrast to other pathogenic strains of the *B. cereus* group, such as *B. anthracis* and *B. thuringiensis*.

The main toxins of pathogenic *B. cereus* (CytK, Hbl, and Nhe) contain secretory signal peptides, suggesting secretion by the general secretory (Sec) pathway (van Wely et al., 2001). In our study, several genes encoding proteins of the Sec-dependent protein export pathway were found in SR52, which is consistent with the likely presence of extracellular cytotoxins in the culture supernatant of SR52. The presence of multiple SPases may enable SR52 to modulate its secretion processing machinery (Bron et al., 1998). Previous studies showed that PlcR is a global regulator that controls the synthesis of many proteins, such as phospholipases, proteases, and toxins, and is important for *B. cereus* virulence (Gohar et al., 2008). The *plcR*-*papR* operon is present in all strains of the *B. cereus* group, and it is associated with point mutations in specific strains (Anderson et al., 2005; Schmidt et al., 2011). In SR52, the *plcR*-*papR* genes bear no mutations, suggesting the existence of a functional PlcR-PapR quorum sensing system in SR52 that may regulate the secretion of various toxins and other proteins (Mignot et al., 2001).

In conclusion, we demonstrated for the first time that a *B. wiedmannii* isolate from the deep-sea hydrothermal field exhibits apparent cytotoxic effects on vertebrate animals and causes acute mortality following artificial inoculation. Consistently, SR52 carries genes encoding various enterotoxins and hemolysins that likely contribute to the lethality of SR52. In addition, compared with its terrestrial homolog, SR52 exhibits unique genomic features possibly associated with adaptation to the deep-sea environment.

## Data Availability Statement

The datasets generated for this study can be found in the GenBank accession number CP032365 and CP032366.

## Ethics Statement

The animal study was reviewed and approved by The Ethics Committee of Institute of Oceanology, Chinese Academy of Sciences.

## Author Contributions

YZ performed part of the toxic studies and analyzed the genome features of SR52. CC characterized the bacteria and performed part of the toxic studies. JZ obtained the deep sea sample and performed part of the toxic studies. HG analyzed some of the data. LS conceived and designed the experiments. YZ and LS wrote the paper.

### Conflict of Interest

The authors declare that the research was conducted in the absence of any commercial or financial relationships that could be construed as a potential conflict of interest.
