# Supplementary material for: Characterization of the Genome Feature and Toxic Capacity of a Bacillus wiedmannii Isolate From the Hydrothermal Field in Okinawa Trough
Source: Front Cell Infect Microbiol. 2019 Oct 25;9:370. doi: 10.3389/fcimb.2019.00370 (PMC6842932; doi:10.3389/fcimb.2019.00370)
Supplement: Supplementary file 1 [file Data_Sheet_1.pdf]

Supplementary data

Table S1. NCBI accession numbers of the16S rRNA genes and whole genome sequences (WGS) used in phylogenetic analyses.

NCBI sequence accession number	Strain	16S rRNA/WGS
KJ812450	*B. pacificus* EB422^{T}	16S rRNA
KJ812449	*B. mobilis* 0711P9-1^{T}	16S rRNA
KJ812420	*B. paranthracis* Mn5^{T}	16S rRNA
KJ812418	*B. proteolyticus* TD42^{T}	16S rRNA
KJ812444	*B. paramycoides* NH24A2^{T}	16S rRNA
KJ812435	*B. tropicus* N24^{T}	16S rRNA
KJ812207	*B. subtilis* subs. *subtilis* DSM 10^{T}	16S rRNA
KJ812440	*B. albus* N35-10-2^{T}	16S rRNA
KJ812415	*B. luti* TD41^{T}	16S rRNA
KJ812430	*B. nitratireducens* 4049^{T}	16S rRNA
BAUY01000093/ BAUY00000000	*B. weihenstephanensis* DSM 11821^{T}	16S rRNA/WGS
FJ416489/ JOTM00000000	*'B. gaemokensis'* BL3-6	16S rRNA/WGS
JN885201/AKCS01000000	*'B. bingmayongensis'* FJAT-13831	16S rRNA/WGS
FJ416490/ JOTN00000000	*'B. manliponensis'* BL4-6	16S rRNA/WGS
AE016877 / NC_004722	*B. cereus* ATCC 14579^{T}	16S rRNA / WGS
AE016879 / NC_003997	*B. anthracis str. Ames*	16S rRNA / WGS
ACNF01000156 /NZ_CM000753	*B. thuringiensis* ATCC 10792^{T}	16S rRNA / WGS
ACMX01000133 / NZ_CM000745	*B. pseudomycoides* DSM 12442^{T}	16S rRNA / WGS
ACMU01000002 / NZ_CM000742	*B. mycoides* DSM 2048^{T}	16S rRNA / WGS
CP000764 / NC_009674.1	*B. cytotoxicus* NVH 391-98^{T}	16S rRNA / WGS
CP006863 / CP006863.1	*B. toyonensis* BCT-7112^{T}	16S rRNA / WGS
KU198626 / SRX1297474	*B. wiedmannii* FSL W8-0169^{T}	16S rRNA / WGS

Table S2. Predicted DNA-DNA hybridization (DDH) between SR52 and representative strains of other *B. cereus* group species.

Isolates	DDH[a]	Model C.I.[b]	Distance	Prob.[c]DDH>= 70%
"*B. bingmayongensis*" FJAT-13831	27.1	24.8 - 29.6%	0.1593	0.03
"*B. manliponensis*" BL4-6	22.8	20.6 - 25.3%	0.1917	0
"*B. gaemokensis*" BL3-6	26.4	24.1 - 28.9%	0.1641	0.02
B. wiedmannii FSL W8-0169[T]	88.4	85.9 - 90.5%	0.0139	95.21
B. thuringiensis ATCC 10792[T]	44.3	41.8 - 46.9%	0.0861	7.21
B. mycoides DSM 2048[T]	40.7	38.2 - 43.2%	0.0972	3.21
B. toyonensis BCT 7112[T]	44.5	41.9 - 47.1%	0.0857	7.43
B. cereus ATCC 14579[T]	44.5	42 - 47.1%	0.0857	7.44
B. cytotoxicus NVH 391-98[T]	25.9	23.6 - 28.4%	0.1675	0.02
B. pseudomycoides DSM 12442[T]	27.4	25.1 - 29.9%	0.1574	0.03
B. weihenstephanensis DSM 11821[T]	40.5	38 - 43.1%	0.0977	3.1
B. anthracis str. Ames	49.5	46.7 - 52.2%	0.064	21.46

[a] DDH represents predicted DNA-DNA Hybridization values calculated using formula 2, which is appropriate for draft genome analysis, as it does not depend on the genome.

[b] C.I. is estimated confidence interval.

[c] Prob. is probability of DDH being equal or above 70%, which is set as a species cut-off

Table S3. Predicted genomic islands in SR52 genome.

GIs-id	Start	End	Size(bp)	G+C content (%)
GIs01	1042441	1050889	8449	35.8
GIs02	1502075	1505471	3397	34
GIs03	1584816	1593011	8196	35.3
GIs04	2397661	2403662	6002	33.9
GIs05	1042441	1050889	31599	31.1
GIs06	2462524	2494122	6184	35.6
GIs07	3058200	3064304	6105	32.6
GIs08	3069534	3080730	11197	33.1
GIs09	3466793	3478234	11442	34.5
GIs10	3682000	3707031	25032	33.2
GIs11	3713547	3725648	12102	41.8
GIs12	3865111	3874875	9765	37.5
GIs13	4210421	4215055	4635	36.2

Table S4. Interspersed and tandem repeats in SR52

Location	Type	Number	Total length (bp)	In location (%)	Average length (bp)
Chromosome/Plasmid	LTR	111/5	11729/367	0.2153/0.2667	107/74
Chromosome/Plasmid	DNA	43/2	2863/161	0.0525/0.1170	67/81
Chromosome/Plasmid	LINE	53/2	3287/304	0.0603/0.2209	62/152
Chromosome/Plasmid	SINE	11/2	916/139	0.0168/0.1010	83/70
Chromosome/Plasmid	RC	4/1	204/29	0.0037/0.0211	51/29
Chromosome/Plasmid	TRF	317/7	50232/369	0.9220/0.2682	158/53

Table S5 Virulence genes of SR52 predicted by blast search of the Virulence Factors of Pathogenic Bacteria database (VFDB). The genes that are absent in *Bacillus wiedmannii* FSL W8-0169 are indicated by ***.**

Gene id	Identity	E value	VFDB name
SR52-GM000243	45.1	1.30E-59	LPS
SR52-GM000283	77.1	0.00E+00	ClpC
SR52-GM000290	40.2	6.50E-20	Polysaccharide capsule
SR52-GM000309	75.4	4.00E-177	EF-Tu
SR52-GM000362	46.3	6.20E-102	Exopolysaccharide
SR52-GM000373	43.3	3.80E-54	IlpA
SR52-GM000384	49.3	2.20E-35	Capsule
SR52-GM000390	41.4	5.00E-114	Oligopeptide-binding protein
SR52-GM000425	40.6	1.90E-36	Capsule
SR52-GM000465	74.7	1.20E-213	GroEL
SR52-GM000507	45.2	1.50E-79	Purine
SR52-GM000536	41.3	7.00E-56	Alginate
SR52-GM000604	45.9	3.10E-08	Cytolysin
SR52-GM000644	40.6	9.10E-64	Type IV pili biosynthesis
SR52-GM000683	49.2	1.30E-120	Heme biosynthesis
SR52-GM000697	42.7	6.60E-42	T4SS effectors
SR52-GM000708*	67.6	7.00E-144	Internalin-like
SR52-GM000709	48.9	3.10E-252	Kappa-toxin (collagenase)
SR52-GM000721	42.5	7.20E-46	PhoP/R
SR52-GM000765	50.7	1.30E-75	achromobactin
SR52-GM000766	45.7	2.60E-71	achromobactin
SR52-GM000767	63.4	1.10E-93	Achromobactin biosynthesis and transport
SR52-GM000787	40.3	6.90E-35	DevR/S
SR52-GM000806	40.3	8.10E-42	RegX3
SR52-GM000814	50	1.10E-33	Capsule
SR52-GM000815	42.9	1.30E-24	Capsule
SR52-GM000827	98	0.00E+00	InhA
SR52-GM000832	99.6	2.40E-168	Phosphatidylcholine-preferring phospholipase C

			(PC-PLC)
SR52-GM000833	97	4.10E-191	SM-PLC
SR52-GM000843	42.7	6.60E-14	Sortase A
SR52-GM000851	46.9	1.50E-09	Cytolysin
SR52-GM000913	41.6	8.30E-28	ABC transporter for dispersin
SR52-GM001024	48.5	6.70E-165	Bcp pili
SR52-GM001044	55.3	3.00E-25	Type VII secretion system
SR52-GM001072	49.3	2.00E-33	Capsule
SR52-GM001162*	40.4	2.30E-61	RTX toxin
SR52-GM001261	40.1	9.50E-66	Heme biosynthesis
SR52-GM001278	62.2	4.40E-121	Lipoate protein ligase A1
SR52-GM001293	40.8	1.10E-33	PhoP/R
SR52-GM001303	66.5	2.70E-163	Isocitrate lyase
SR52-GM001327	44.2	4.80E-116	Catalase
SR52-GM001344	45.8	1.50E-205	ClpC
SR52-GM001357	43.8	8.20E-125	Oligopeptide-binding protein
SR52-GM001361	48.9	2.30E-39	Capsule
SR52-GM001363	45.6	4.20E-132	Oligopeptide-binding protein
SR52-GM001384	48.3	3.80E-57	GPL locus
SR52-GM001390	46.8	1.70E-53	Capsule
SR52-GM001391	46.4	2.40E-44	Capsular polysaccharide
SR52-GM001392*	51.5	7.60E-94	LPS
SR52-GM001393*	40.1	4.00E-54	Capsule
SR52-GM001433	50.9	2.50E-07	Cytolysin
SR52-GM001449	95.1	0.00E+00	Immune inhibitor A metalloprotease
SR52-GM001451	48.9	8.60E-61	Trehalose-recycling ABC transporter
SR52-GM001465	41.1	4.80E-47	RegX3
SR52-GM001481	44.2	1.40E-50	Polar flagella
SR52-GM001488	48.3	1.30E-62	Trehalose-recycling ABC transporter
SR52-GM001496*	43.8	3.80E-49	Internalin-like
SR52-GM001540	50.8	2.70E-154	D-alanine-polyphosphoribitol ligase
SR52-GM001571	44.8	2.70E-36	Leucine synthesis

SR52-GM001576	40.9	1.40E-35	LPS O-antigen (P. aeruginosa)
SR52-GM001578	44.8	7.90E-62	LPS
SR52-GM001587	43.3	5.80E-100	Lysine synthesis
SR52-GM001591	48.7	5.70E-47	Phytotoxin phaseolotoxin
SR52-GM001634	48.5	1.30E-47	Mn] superoxide dismutase [Iron-cofactored SOD
SR52-GM001642	41	2.20E-42	LisR/LisK
SR52-GM001675	44.9	7.80E-16	histone-like protein (Hlp)/laminin-binding protein (LBP)
SR52-GM001680	52.7	8.80E-37	Nucleoside diphosphate kinase
SR52-GM001706	44.4	1.20E-53	Pantothenate synthesis
SR52-GM001707	56.1	2.70E-34	Pantothenate synthesis
SR52-GM001787	97.1	4.30E-146	peritrichous flagella
SR52-GM001788	97.3	2.40E-94	peritrichous flagella
SR52-GM001789	97.5	5.50E-61	peritrichous flagella
SR52-GM001790	90.3	0.00E+00	peritrichous flagella
SR52-GM001791	92.3	5.30E-265	peritrichous flagella
SR52-GM001794	95	2.70E-142	peritrichous flagella
SR52-GM001798	96.3	1.60E-235	peritrichous flagella
SR52-GM001799	96.9	1.90E-152	peritrichous flagella
SR52-GM001800	92	3.30E-215	peritrichous flagella
SR52-GM001801	99.2	2.40E-64	peritrichous flagella
SR52-GM001803	94.1	1.70E-66	peritrichous flagella
SR52-GM001804	98.5	5.90E-72	peritrichous flagella
SR52-GM001805	96	5.60E-48	peritrichous flagella
SR52-GM001806	95.1	2.70E-242	peritrichous flagella
SR52-GM001807	97.6	4.80E-176	peritrichous flagella
SR52-GM001808	98.7	7.80E-85	peritrichous flagella
SR52-GM001809	97.5	8.60E-242	peritrichous flagella
SR52-GM001812	93.2	1.40E-95	peritrichous flagella
SR52-GM001813	83.4	3.10E-193	peritrichous flagella
SR52-GM001817	97	2.70E-162	peritrichous flagella
SR52-GM001819*	90.6	3.90E-128	peritrichous flagella

SR52-GM001820	54	3.80E-102	peritrichous flagella
SR52-GM001821*	56.8	4.90E-108	peritrichous flagella
SR52-GM001822	42.4	2.00E-20	T3SS
SR52-GM001823	97.4	1.80E-36	peritrichous flagella
SR52-GM001824	98.5	6.20E-184	peritrichous flagella
SR52-GM001825	98.3	1.90E-58	peritrichous flagella
SR52-GM001827	100	1.10E-129	peritrichous flagella
SR52-GM001828	100	5.60E-34	peritrichous flagella
SR52-GM001829	99.6	8.80E-130	peritrichous flagella
SR52-GM001830	47.9	2.90E-83	<beta>-GlcNAc
SR52-GM001831	98.3	0.00E+00	peritrichous flagella
SR52-GM001832	97	8.80E-218	peritrichous flagella
SR52-GM001833	97.7	1.50E-137	peritrichous flagella
SR52-GM001843	52.6	9.20E-156	Bcp pili
SR52-GM001844	62.6	3.80E-94	Bcp pili
SR52-GM001851	47.8	1.50E-52	Trehalose-recycling ABC transporter
SR52-GM001930	52.3	2.20E-105	Trehalose-recycling ABC transporter
SR52-GM001948	41.2	1.10E-113	Oligopeptide-binding protein
SR52-GM001986	40	1.50E-118	Oligopeptide-binding protein
SR52-GM002001	97.4	5.70E-205	Nonhemolytic enterotoxin NHE
SR52-GM002002	99.5	3.40E-200	Nonhemolytic enterotoxin NHE
SR52-GM002003	93.9	4.70E-182	Nonhemolytic enterotoxin NHE
SR52-GM002098	40.7	3.80E-36	MprA/B
SR52-GM002104	41.9	3.50E-39	PdgA
SR52-GM002112	40	2.40E-50	Polysaccharide capsule
SR52-GM002114	40.5	2.50E-38	LisR/LisK
SR52-GM002120	91.7	0.00E+00	petrobactin
SR52-GM002121	90.3	0.00E+00	petrobactin
SR52-GM002122	94.4	2.10E-229	petrobactin
SR52-GM002123	91.7	1.40E-36	petrobactin
SR52-GM002124	96	9.50E-185	petrobactin
SR52-GM002125	95	8.50E-158	petrobactin

SR52-GM002173	44.2	1.30E-128	Oligopeptide-binding protein
SR52-GM002227	55.3	3.90E-25	Type VII secretion system
SR52-GM002231	46.2	4.20E-12	Type VII secretion system
SR52-GM002233	43.8	0.00E+00	Type VII secretion system
SR52-GM002283	52.5	0.00E+00	Nitrate reductase
SR52-GM002284	57.7	1.80E-179	Nitrate reductase
SR52-GM002286	46.2	6.60E-58	Nitrate reductase
SR52-GM002341	50	4.00E-57	LisR/LisK
SR52-GM002351	52.6	1.70E-69	LOS
SR52-GM002369	99.1	7.40E-117	Hemolysin III
SR52-GM002402	44.5	2.80E-51	Trehalose-recycling ABC transporter
SR52-GM002420	45.5	3.20E-64	HIS-2
SR52-GM002477	48	1.70E-59	chrysobactin
SR52-GM002478	40.6	8.80E-84	Vibriobactin biosynthesis
SR52-GM002479	58.3	3.10E-180	Pyochelin
SR52-GM002480	48.5	3.60E-74	chrysobactin
SR52-GM002482	65.6	8.30E-20	Mycobactin
SR52-GM002486	46.1	5.20E-16	histone-like protein (Hlp)/laminin-binding protein (LBP)
SR52-GM002515	95.7	1.60E-219	Hemolytic enterotoxin HBL
SR52-GM002516	99.3	5.60E-195	Hemolytic enterotoxin HBL
SR52-GM002517	95.7	4.40E-202	Hemolytic enterotoxin HBL
SR52-GM002518	86.5	3.00E-232	HBL
SR52-GM002519	41.7	1.80E-130	OatA
SR52-GM002625	41.1	3.70E-13	Capsule
SR52-GM002651	42.3	4.50E-48	VirR/VirS
SR52-GM002653	40.2	1.10E-34	Trehalose-recycling ABC transporter
SR52-GM002662	78.7	1.30E-182	Hemolytic enterotoxin HBL
SR52-GM002663	84.2	1.80E-169	HBL
SR52-GM002664	70.9	9.80E-139	Hemolytic enterotoxin HBL
SR52-GM002672	46.2	2.40E-40	M. catarrhalis adherence protein (McaP)
SR52-GM002718	41.3	4.90E-42	BfmRS

SR52-GM002729	91.1	5.80E-291	Bcp pili
SR52-GM002730	86.8	1.00E-131	Bcp pili
SR52-GM002731*	94.3	2.50E-177	Bcp pili
SR52-GM002732*	81.4	3.60E-178	Bcp pili
SR52-GM002773	43.6	2.40E-52	VirR/VirS
SR52-GM002786	40.2	3.10E-71	Lipase
SR52-GM002803*	44	8.30E-17	Capsule
SR52-GM002885	40.1	2.90E-39	RegX3
SR52-GM002927	52.7	1.90E-77	thioquinolobactin
SR52-GM002928	41.4	8.80E-43	thioquinolobactin
SR52-GM002934	40.3	2.00E-66	Flagella
SR52-GM002943	41.1	3.50E-65	PDH-B
SR52-GM002960	68.9	1.50E-73	ClpP
SR52-GM002965*	44.2	2.40E-40	M. catarrhalis adherence protein (McaP)
SR52-GM003007	45.2	3.10E-68	ND
SR52-GM003022	40.7	1.00E-111	Oligopeptide-binding protein
SR52-GM003144*	42.8	2.20E-69	Flagella
SR52-GM003173	96.1	0.00E+00	InhA1, InhA2, InhA3
SR52-GM003201	40.1	3.50E-76	Catalase
SR52-GM003266	41.1	6.10E-26	PDIM (phthiocerol dimycocerosate) and PGL (phenolic glycolipid) biosynthesis and transport
SR52-GM003398	46.3	7.10E-54	MprA/B
SR52-GM003585	42.4	4.20E-07	Cytolysin
SR52-GM003618	61.3	2.90E-96	O-antigen
SR52-GM003619	45.7	6.40E-94	O-antigen
SR52-GM003668*	42.5	5.10E-47	RegX3
SR52-GM003681	54	1.30E-61	PDIM (phthiocerol dimycocerosate) and PGL (phenolic glycolipid) biosynthesis and transport
SR52-GM003767	42.2	2.20E-47	VirR/VirS
SR52-GM003774	48.1	2.40E-252	Kappa-toxin (collagenase)
SR52-GM003775	46.2	6.40E-23	Magnesium transport
SR52-GM003820	42.3	2.00E-49	Polar flagella

SR52-GM003850	47.9	5.60E-140	Oligopeptide-binding protein
SR52-GM003851	45.2	1.70E-133	Oligopeptide-binding protein
SR52-GM003852	45.8	4.60E-134	Oligopeptide-binding protein
SR52-GM003869	44.5	2.40E-68	Serine protease
SR52-GM003870	44.4	1.50E-48	RegX3
SR52-GM003970	59.2	3.60E-124	LPS
SR52-GM003971	43.5	1.30E-14	LPS
SR52-GM003972	62.9	1.20E-65	Capsular polysaccharide
SR52-GM003985*	65.2	1.10E-190	Polysaccharide capsule
SR52-GM003986	43.8	7.70E-37	Polysaccharide capsule
SR52-GM004049	43.5	4.60E-97	Glutamine synthesis
SR52-GM004072	41.6	1.80E-13	histone-like protein (Hlp)/laminin-binding protein (LBP)
SR52-GM004074	52.6	3.00E-171	Copper exporter
SR52-GM004079	50.4	2.00E-66	ABC transporter
SR52-GM004096*	46.4	4.90E-67	Pse5Ac7Ac
SR52-GM004109	93.9	3.20E-174	Phosphatidylinositol-specific phospholipase C (PI-PLC)
SR52-GM004174	47.7	9.40E-58	Capsule
SR52-GM004175	62.7	3.40E-84	Capsule
SR52-GM004202	59.5	2.60E-16	LPS
SR52-GM004203	51.7	1.40E-60	Polar flagella
SR52-GM004212	48.4	2.50E-53	Capsule
SR52-GM004215	49.4	9.80E-65	Serine-threonine phosphatase
SR52-GM004227	50.3	7.00E-159	Fibronectin-binding protein
SR52-GM004241	47.4	4.70E-281	Pyrimidine biosynthesis
SR52-GM004242	43.9	3.80E-81	Pyrimidine biosynthesis
SR52-GM004248	57	2.70E-44	Lipoprotein-specific signal peptidase II
SR52-GM004285	42	2.30E-30	LPS
SR52-GM004329	46.1	2.60E-86	PDH-B
SR52-GM004354	40.6	8.70E-127	type IV pili
SR52-GM004377*	53.4	3.70E-105	Trehalose-recycling ABC transporter

SR52-GM004426	44.5	4.90E-24	Dot/Icm
SR52-GM004522	45.9	4.10E-79	PDH-B
SR52-GM004529	43.2	3.90E-63	lateral flagella
SR52-GM004538	58.1	6.20E-84	Hemolysin
SR52-GM004667	46.9	9.20E-61	xcp secretion system
SR52-GM004700	51.3	1.40E-53	SodB
SR52-GM004717	63	1.80E-99	Sigma A
SR52-GM004738	59.6	1.10E-186	MOMP
SR52-GM004792	48.1	1.80E-59	NEAT-type hemophore-mediated heme uptake system
SR52-GM004793	40.2	9.40E-58	direct heme uptake system
SR52-GM004796	74.5	0.00E+00	Listeria adhesion protein
SR52-GM004832	44.6	2.70E-179	(p)ppGpp synthesis and hydrolysis
SR52-GM004865*	48	1.50E-59	VirR/VirS
SR52-GM004886	57.9	5.50E-140	Heme biosynthesis
SR52-GM004887	48.3	1.50E-81	Heme biosynthesis
SR52-GM004889	46.6	9.00E-68	Heme biosynthesis
SR52-GM004898	46.6	3.10E-103	Trigger factor
SR52-GM004904	48	5.30E-40	LOS
SR52-GM004913	42.4	1.10E-06	DevR/S
SR52-GM004923	85.3	2.00E-113	peritrichous flagella
SR52-GM004924	96.2	1.60E-137	peritrichous flagella
SR52-GM004949	41.1	1.40E-52	RegX3
SR52-GM004953	91.6	1.30E-53	NEAT-type hemophore-mediated heme uptake system
SR52-GM004954	96.5	2.00E-137	NEAT-type hemophore-mediated heme uptake system
SR52-GM004955	97.7	8.60E-141	NEAT-type hemophore-mediated heme uptake system
SR52-GM004956	98.8	4.40E-174	NEAT-type hemophore-mediated heme uptake system
SR52-GM004957	95.9	1.70E-153	NEAT-type hemophore-mediated heme uptake system
SR52-GM004958	76.9	0.00E+00	NEAT-type hemophore-mediated heme uptake system
SR52-GM004959	95.4	9.80E-76	NEAT-type hemophore-mediated heme uptake system
SR52-GM004960	81.4	5.20E-92	NEAT-type hemophore-mediated heme uptake system
SR52-GM004995	48.9	8.40E-83	Streptococcal plasmin receptor/GAPDH
SR52-GM005001	44.5	7.70E-51	RegX3

SR52-GM005038	45.2	9.20E-10	Cytolysin
SR52-GM005098	40.3	4.20E-46	RegX3
SR52-GM005194	47	7.20E-19	RicA
SR52-GM005218	40.2	6.70E-37	ABC transporter
SR52-GM005252	40.5	4.10E-41	RegX3
SR52-GM005271*	40.6	2.90E-37	BfmRS
SR52-GM005279*	43.9	2.00E-51	VirR/VirS
SR52-GM005300	41.5	1.00E-43	Enterobactin synthesis
SR52-GM005324	43.7	5.60E-19	Magnesium transport
SR52-GM005351	54.8	6.40E-29	GtcA
SR52-GM005352	46.7	1.20E-81	LPS
SR52-GM005353	82.8	2.10E-138	Polysaccharide capsule
SR52-GM005356	50.7	1.20E-56	Capsule
SR52-GM005470	43.1	3.90E-07	Cytolysin
SR52-GM005524	98.4	1.50E-290	Anthrolysin O
SR52-GM005526	97.6	1.30E-136	petrobactin
SR52-GM005527	97.7	1.50E-183	petrobactin
SR52-GM005528	97.9	2.80E-176	petrobactin
SR52-GM005529	94	2.70E-174	petrobactin
SR52-GM005530	41.6	1.10E-46	LisR/LisK
SR52-GM005541	75.2	3.60E-184	Fibronectin-binding protein
SR52-GM005545	55	5.70E-92	Streptococcal plasmin receptor/GAPDH
SR52-GM005556	78.2	3.30E-84	ClpP
SR52-GM005567	60.1	8.10E-89	Lipoprotein diacylglyceryl transferase
SR52-GM005578	48.3	1.10E-08	Cytolysin
SR52-GM005606	63.1	3.10E-131	Capsule
SR52-GM005607	56.1	1.40E-135	Polysaccharide capsule
SR52-GM005661	48.4	6.80E-10	Cytolysin
SR52-GM005667	40.2	3.20E-43	MprA/B
SR52-GM005668	98.5	4.30E-193	Polysaccharide capsule
SR52-GM005669*	95.6	1.40E-144	Polysaccharide capsule
SR52-GM005670	93.4	1.70E-159	Polysaccharide capsule

SR52-GM005674*	63.3	2.60E-161	LPS
SR52-GM005677*	71.4	9.60E-157	Capsule
SR52-GM005679*	71.6	7.20E-138	Capsule
SR52-GM005680*	45.4	1.50E-91	Capsule
SR52-GM005682*	71	7.50E-76	Capsule
SR52-GM005683	94.5	0.00E+00	Polysaccharide capsule
SR52-GM005685*	94.1	5.60E-140	Polysaccharide capsule
SR52-GM005686*	97.9	4.60E-125	Polysaccharide capsule
SR52-GM005687*	97.6	3.20E-124	Polysaccharide capsule
SR52-GM005688*	96.4	1.80E-118	Polysaccharide capsule
SR52-GM005689	45.7	4.10E-31	LPS
SR52-GM005764	94.7	3.80E-153	PlcR
SR52-GM005795	51.4	1.90E-69	exochelin
SR52-GM005796*	40.3	7.30E-55	staphyloferrin A
SR52-GM005848	43.8	3.40E-70	Methionine sulphoxide reductase
SR52-GM005858	42.7	2.60E-42	SodB
SR52-GM005861	83.6	3.40E-169	Polysaccharide capsule
SR52-GM005862	96.8	2.20E-116	Hemolysin III homolog
SR52-GM005871	43.5	6.70E-68	Serine protease
SR52-GM005876	49.6	3.70E-58	RegX3
